# Congruency effects in dot comparison tasks: convex hull is more important than dot area

**DOI:** 10.1080/20445911.2016.1221828

**Published:** 2016-08-31

**Authors:** Camilla Gilmore, Lucy Cragg, Grace Hogan, Matthew Inglis

**Affiliations:** ^a^ Mathematics Education Centre, Loughborough University, Loughborough, UK; ^b^ School of Psychology, University of Nottingham, Nottingham, UK; ^c^ Department of Mathematical Sciences, Loughborough University, Loughborough, UK

**Keywords:** Dot comparison, congruency effects, numerical cognition, magnitude representations

## Abstract

The dot comparison task, in which participants select the more numerous of two dot arrays, has become the predominant method of assessing Approximate Number System (ANS) acuity. Creation of the dot arrays requires the manipulation of visual characteristics, such as dot size and convex hull. For the task to provide a valid measure of ANS acuity, participants must ignore these characteristics and respond on the basis of number. Here, we report two experiments that explore the influence of dot area and convex hull on participants’ accuracy on dot comparison tasks. We found that individuals’ ability to ignore dot area information increases with age and display time. However, the influence of convex hull information remains stable across development and with additional time. This suggests that convex hull information is more difficult to inhibit when making judgements about numerosity and therefore it is crucial to control this when creating dot comparison tasks.

Over the past decade, researchers in mathematical cognition have become increasingly interested in discovering the basic representations and processes that are associated with successful mathematics performance. This interest stems, in part, from a belief that discovering the cognitive underpinnings of mathematical performance will highlight avenues for the development of future educational interventions. One area to receive particular focus is the role of basic numerical representations. It has been proposed that adults and children are capable of representing magnitude information within an Approximate Number System (ANS; Barth et al., 2006) and individual differences in the acuity of this system are related to individual differences in mathematics achievement (Halberda, Mazzocco, & Feigenson, 2008).

According to the ANS theory, individuals are capable of representing the approximate number of items (*n*) in a set, without counting them. These representations are approximate and vary according to a normal distribution with mean *n* and standard deviation *wn*, where *w* is the Weber fraction, which varies from one individual to another. Individuals with a smaller *w* form more precise representations of numerical magnitude and, it is proposed, have more success in learning mathematics (Halberda et al., 2008).

The predominant task used to assess ANS acuity is the dot comparison task. This involves presenting pairs of dot arrays and asking participants to select the more numerous array. The ratio between the numerosities of the two arrays is varied, which consequently affects the difficulty of each trial; trials with numerosity ratios closer to 1 are more difficult. Participants’ accuracy on this task is used to index the acuity of their underlying magnitude representations and, in some studies, estimates are computed of participants’ Weber fractions (e.g. Halberda et al., 2008). Some studies have shown significant correlations between performance on a dot comparison task and mathematics achievement (see meta-analyses by Chen & Li, 2014; Fazio, Bailey, Thompson, & Siegler, 2014; Schneider et al., 2016), although a substantial body of conflicting evidence also exists (see review by De Smedt, Noël, Gilmore, & Ansari, 2013).

Recent efforts to understand this conflicting evidence has highlighted that dot comparison task performance does not provide a pure measure of ANS acuity. It has been shown that performance on dot comparison tasks, and hence estimates of ANS acuity, are influenced by the numerosity of the dot arrays (Clayton & Gilmore, 2015), the method of presentation (i.e. sequential vs. simultaneous; Price, Palmer, Battista, & Ansari, 2012), the display time (Inglis & Gilmore, 2013) and the measure of performance used (e.g. accuracy or RT; Inglis & Gilmore, 2014). In particular, the visual characteristics of dot arrays have been shown to have substantial impact on participants’ performance (Clayton, Gilmore, & Inglis, 2015; DeWind & Brannon, 2016; Gebuis & Reynvoet, 2012; Smets, Sasanguie, Szücs, & Reynvoet, 2015). Studies have shown that performance on dot comparison tasks is dependent on how the visual characteristics of arrays are controlled and, in particular, how many visual cues are controlled (Gebuis & Reynvoet, 2012; Smets et al., 2015). Researchers use a variety of methods to control for visual characteristics in the construction of dot arrays (e.g. Gebuis & Reynvoet, 2011; Halberda et al., 2008; Pica, Lemer, Izard, & Dehaene, 2004; see Clayton et al., 2015 for examples of stimuli from different methods). Typically this involves the creation of congruent trials, in which one or more visual cues are positively correlated with numerosity, and incongruent trials, in which one or more visual cues are negatively correlated with numerosity. The visual cues which may be controlled include dot area (either cumulative surface area or average dot area, which are highly correlated), density or convex hull (i.e. the smallest contour around the array of dots). Several studies have demonstrated that these visual cues can influence numerosity judgements even when efforts are made to control these (Barth et al., 2006; Gebuis, Cohen Kadosh, De Haan, & Henik, 2009; Gilmore et al., 2013; Nys & Content, 2012). According to some accounts, numerosity judgements are made solely on the basis of these visual characteristics without any role for a specific numerosity representation system (Gebuis & Reynvoet, 2012).

One explanation for why the visual characteristics of dot arrays affects magnitude comparison judgements focuses on the role of inhibition (Gilmore et al., 2013; Szűcs, Nobes, Devine, Gabriel, & Gebuis, 2013). It has been suggested that to perform successfully on incongruent dot comparison trials requires inhibition, in order to suppress a response based on the salient visual characteristics, in favour of a response based on the relative numerosities of the arrays. In support of this proposal, evidence has shown that participants are more accurate on congruent, compared with incongruent, dot comparison trials (e.g. Barth et al., 2006; Gebuis et al., 2009; Gilmore et al., 2013; Nys & Content, 2012), the difference in performance on congruent and incongruent trials is related to inhibition skill (Gilmore, Keeble, Richardson, & Cragg, 2015) and the relationship between dot comparison performance and mathematics achievement is accounted for by inhibition skill (Fuhs & McNeil, 2013; Gilmore et al., 2013).

One argument against this proposal is that some studies fail to find evidence of congruency effects (e.g. Odic, Libertus, Feigenson, & Halberda, 2013). In these studies, participants perform as accurately on congruent as on incongruent trials. However, as described above, studies differ in the ways in which they control for visual characteristics as well as other features of the dot comparison task (e.g. display time). These factors are likely to affect the nature of congruency effects. The size of congruency effects have been shown to be dependent on the type and number of visual cues that are manipulated (Gebuis & Reynvoet, 2012). In particular, adults’ performance on dot comparison tasks appears to be influenced more by convex hull than by cumulative surface area or average dot size (Clayton et al., 2015).

There is also some evidence that the influence of visual cues changes across development. While adults’ dot comparison performance is only influenced by convex hull (Clayton et al., 2015), numerosity judgements of children aged 7–9 years old are influenced by both the convex hull and the cumulative surface area of dots in an array (Clayton & Gilmore, 2015). This appears to indicate that convex hull information is more difficult to inhibit than cumulative surface area information, when making numerosity judgements. If this is the case then it is possible that adults will also be influenced by dot area information if the task is made more difficult, for example, by reducing the display time.

Here, we present two experiments that explore congruency effects on dot comparison tasks to investigate the types of visual information which influence participants’ numerosity judgements. We focused on the role of convex hull and dot area information. Information about average dot diameter and array density were highly correlated with dot area information and thus cannot be differentiated (Gebuis & Reynvoet, 2012). In Experiment 1, we investigated the effect of convex hull information and dot area information on the dot comparison performance of participants aged from 5–6 years to adult. We predicted that dot area information would have a decreasing influence on performance with increasing age, whereas the influence of convex hull information would remain stable across ages. In Experiment 2, we investigated how changes in display time would affect the influence of visual cues on adults’ dot comparison performance. We predicted that convex hull information would have a consistent influence on numerosity judgements regardless of display time, whereas dot area information would influence performance more with reduced display time. In these experiments, we focus on the influence of convex hull information and dot area information.

The goal of the experiments was to help us better understand the source of congruency effects as well as the factors, both of the task and the participants, which influence their presence. These are important questions for both methodological and theoretical reasons. Methodologically, it is important to understand how task characteristics influence congruency effects in order to appropriately design tasks, as well as to inform on the extent to which findings across studies can be compared. Theoretically, it is important to understand why congruency effects arise in order to reveal the underlying processes involved in making numerosity judgements. This is essential to be able to make sense of the conflicting evidence surrounding the relationship between the acuity of numerical representations and mathematics achievement.

## Experiment 1

### Method

#### Participants

368 participants took part in Experiment 1 comprising 75 children aged 5–6 years (*M* = 6.17 years, SD = 0.36; 41 male), 84 children aged 8–9 years (*M* = 8.89, SD = 0.28; 38 male), 67 children aged 11–12 years (*M* = 12.23, SD = 0.37; 35 male), 67 children aged 13–14 years (*M* = 14.23, SD = 0.30; 30 male), and 75 young adults (*M* = 21.28, SD = 1.69; 27 male).

#### Stimuli and apparatus

Participants completed a dot comparison task as part of a larger battery of mathematical and general cognitive tasks. The dot comparison task was presented on a laptop computer and consisted of 6 practice trials and 80 experimental trials. The ratio between the numerosity of the presented arrays was 0.5, 0.6, 0.7, or 0.8 and the numerosities ranged from 5 to 28.

The dot arrays were constructed following the method of Gebuis and Reynvoet (2011) which resulted in four types of trial: congruent for both dot area and convex hull; congruent for dot area and incongruent for convex hull; incongruent for dot area and congruent for convex hull; incongruent for both dot area and convex hull. After creating the arrays, we calculated the cumulative surface area of the dots for each array and used the Graham Scan algorithm (Graham, 1972) to obtain values of the convex hull for each array. We then calculated the convex hull ratio and cumulative dot area ratio for each trial (see Table 1). We also calculated the average dot area ratio and density ratio for each trial and found that, as described by Gebuis and Reynvoet (2012), they were highly correlated with cumulative dot area ratio (*r* = .97 and .80, respectively).

**Table 1.** Ratio values (correct:incorrect) for the convex hull and cumulative dot area for stimuli used in Experiments 1 and 2. Ratios less than one indicate incongruent trials and ratios above one indicate congruent trials.

	Experiment 1		Experiment 2	
	Mean	Range	Mean	Range
Convex hull	1.48	0.35–7.35	1.56	0.50–4.04
Dot area	4.55	0.25–15.11	6.88	0.23–27.12

#### Procedure

On each trial of the dot comparison task two dot arrays were presented side-by-side on the screen and participants’ task was to select the more numerous array and press a key that corresponded to the appropriate side of the screen. Due to evidence of the impact of display time on dot comparison performance (Inglis & Gilmore, 2013), the dot arrays were presented for a fixed time of 1000 ms before disappearing and being replaced by a question mark. Participants could only respond after the dot arrays disappeared.

### Results and discussion

Accuracy scores were used to index performance on the task as they are strongly correlated with *w* estimates but have superior reliability (Inglis & Gilmore, 2014). The effect of the characteristics of the arrays on participants’ accuracy was analysed via a by-items multi-level linear model with items as a Level 2 variable and the mean accuracy for each age group as repeated-measures variable within each item. The purpose of a by-items analysis is to explore the effects of item characteristics on accuracy, averaging across participants. For each trial we calculated log ratios for the number of dots, the convex hull and the total dot area of each array. These were *z*-transformed before analysis.

We first verified that the fit of the model was improved by allowing intercepts to vary across items compared with a model with only fixed effects, *χ* ^2^(1) = 75.79, *p* < .001, but allowing slopes to vary across items did not further improve the model. We then explored the effects of item characteristics (numerical ratio, convex hull ratio, and dot area ratio) and age (*z*-transformed group mean age) by including all main effects and interactions as fixed-effects predictors. Our prediction was that there would be a significant three-way interaction between convex hull ratio, dot area ratio, and age.

**Figure 1.**
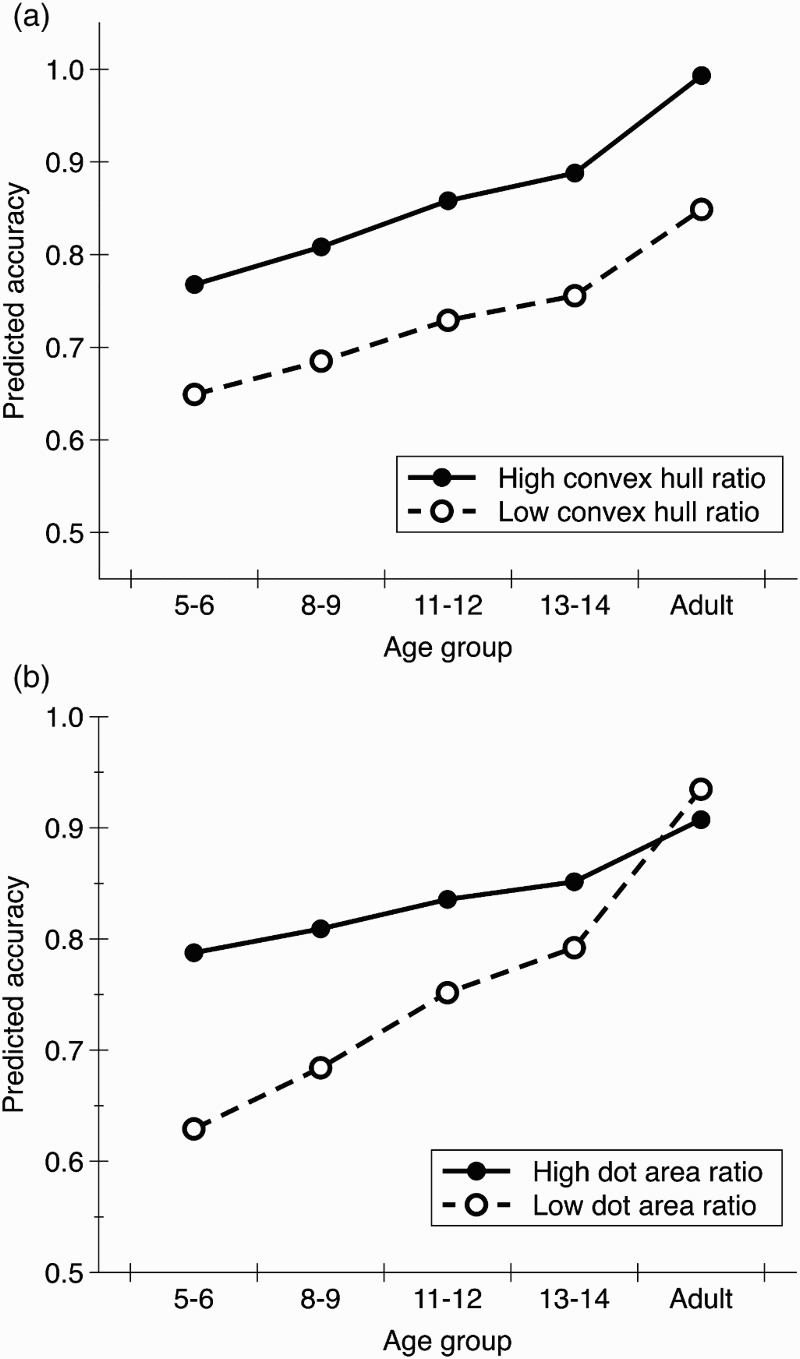
Predicted accuracy scores for each age group with (a) high (+1 *SD*) or low (−1 *SD*) values of convex hull ratio and mean values of dot area ratio and (b) high (+1 *SD*) or low (−1 *SD*) values of dot area ratio and mean values of convex hull ratio. In each case mean values of numerical ratio are used.

In the final model there were significant main effects of convex hull ratio, *b* = .06, *t*(80) = 9.85, *p* < .001, dot area ratio, *b* = .04, *t*(80) = 6.10, *p* < .001, numerical ratio, *b* = .05, *t*(80) = 8.14, *p* < .001, and age, *b* = .07, *t*(320) = 22.44, *p* < .001. There were also significant interactions: age × dot area ratio, *b* = −.03, *t*(320) = −9.65, *p* < .001, age × numerical ratio, *b* = .01, *t*(320) = 2.86, *p* = .005, convex hull ratio × numerical ratio, *b* = −.03, *t*(80) = −5.01, *p* < .001, and a marginally significant interaction between age × convex hull ratio × numerical ratio, *b* = −.01, *t*(320) = −2.03, *p* = .044. Importantly, the predicted three-way interaction between convex hull ratio, dot area ratio, and age was significant, *b* = .01, *t*(320) = 3.35, *p* = .001. Other effects were non-significant. Figure 1 displays predicted accuracy scores for high and low values of convex hull and dot area ratio for each age group.

To explore the three-way interaction between convex hull ratio, dot area ratio and age, we investigated the relationship between accuracy and convex hull ratio and the relationship between accuracy and dot area ratio for each age group separately. There was a significant correlation between convex hull ratio and accuracy for each age group (Year 1: *r* = .57, *p* < .001, Year 4: *r* = .64, *p* < .001, Year 7: *r* = .66, *p* < .001, and Year 9: *r* = .68, *p* < .001, Adults: *r* = .63, *p* < .001), and the strength of this correlation remained stable across age groups. In contrast, the correlation between dot area ratio and accuracy was significant for each of the groups of children (Year 1: *r* = .72, *p* < .001, Year 4: *r* = .61, *p* < .001, Year 7: *r* = .38, *p* = .001, and Year 9: *r* = .33, *p* = .003) but was not significant for the adult participants, *r* = .13, *p* = .253.^1^ 1 This pattern of relationships remains the same if partial correlations are used. The correlation between convex hull ratio and accuracy controlling for dot area ratio is significant and stable across all age groups while the correlation between dot area ratio and accuracy controlling for convex hull ratio is significant only for the groups of children and decreases in strength with increasing age. Moreover the correlation between dot area ratio and accuracy was significantly higher for children in Year 1 than children in Years 7 and 9 and adults and was significantly higher for children in Year 4 than children in Year 9 or adults (Fisher’s *r*-to-*z* transformation, all *p*s < .05).^2^ 2 The same pattern is observed if performance is explored using simpler congruency effects. For this analysis all trials were categorized as congruent or incongruent for dot area and convex hull. An ANOVA with age group as between-groups variable and dot area congruency and convex hull congruency as repeated-measures variables found a significant three-way interaction (*F*(4,363) = 7.0, *p* < .001). Dot area and convex hull congruency effects were calculated for each participant (congruent accuracy minus incongruent accuracy), and there was a significant negative correlation between dot area congruency effect and age (*r_s_* = −.352, *p* < .001), but no correlation between convex hull congruency effect and age (*r_s_* = −.004, *p* = .932)


This pattern of results suggests that it is more difficult to overcome convex hull information than dot area information. Across all age groups, we found that participants were significantly more accurate when the convex hull ratio was larger and the strength of this relationship was similar across all age groups. In contrast, there was a stronger relationship between dot area ratio and accuracy for younger children than older children or adults. Indeed adults performed no more accurately on trials with a larger dot area ratio than trials with a smaller dot area ratio. This raises the question of whether adults are able to ignore dot area information in all situations or whether they would also show a relationship between dot area ratio and accuracy under particular conditions, for example, if the task were more difficult.

One way to manipulate the difficulty of the dot comparison task is to alter the stimuli display duration (Inglis & Gilmore, 2013). The present study employed a relatively long 1000 ms display time, which was necessary so that the task was suitable for all age groups from 5 years old. In Experiment 2, with adult participants, we varied the period of time for which dot arrays were displayed. In line with the results of Experiment 1, we expected that convex hull ratio would have a stable influence on performance across display times while dot area ratio would have a bigger influence on performance with shorter display times.

## Experiment 2

### Method

#### Participants

Twenty participants (11 male) took part in Experiment 2 with ages ranging from 20 to 59 years. The participants were adults recruited from the Mathematics Education Centre’s participant pool (predominantly staff and students at Loughborough University).

#### Stimuli and apparatus

Participants completed a dot comparison task presented on a laptop computer. The task consisted of 3 blocks of 80 trials each with different display times for each block (16 ms, 300 ms, or 2400 ms). The ratio between the numerosity of the presented arrays was 0.5, 0.6, 0.7, or 0.8 and the numerosities ranged from 5 to 21.

As in Experiment 1, the dot arrays were constructed following the method of Gebuis and Reynvoet (2011) and we again calculated the convex hull ratio and the cumulative dot area ratio for each trial. We also calculated the average dot area ratio and density ratio for each trial and again found that they were highly correlated with cumulative dot area ratio (*r* = .97 and .79, respectively).

#### Procedure

On each trial of the dot comparison task, participants saw a fixation point for 1000 ms followed by two dot arrays presented side-by-side on the screen. Participants’ task was to select the more numerous array and press a key that corresponded to the appropriate side of the screen. Participants completed three blocks made up of the same 80 trials. The blocks differed in terms of the presentation time of the arrays: 16, 300, or 2400 ms. After the appropriate presentation time, the arrays were replaced by a visual mask and a question mark appeared in the centre of the screen. Participants could only respond after the dot arrays disappeared. The order of blocks was counterbalanced across participants.

### Results and discussion

The effect of the visual characteristics of the arrays on accuracy was analysed via a by-items multiple linear regression with accuracy as dependent variable. Predictors in the model included the numerical ratio, convex hull ratio, and dot area ratio of the two arrays in each trial as well as display time for each trial. Log ratios were used and all variables were *z*-transformed before computing interaction terms. All interaction terms (2-way, 3-way, and 4-way) were also included in the model. Our prediction was that there would be a significant interaction between convex hull ratio, dot area ratio, and display time.

**Figure 2.**
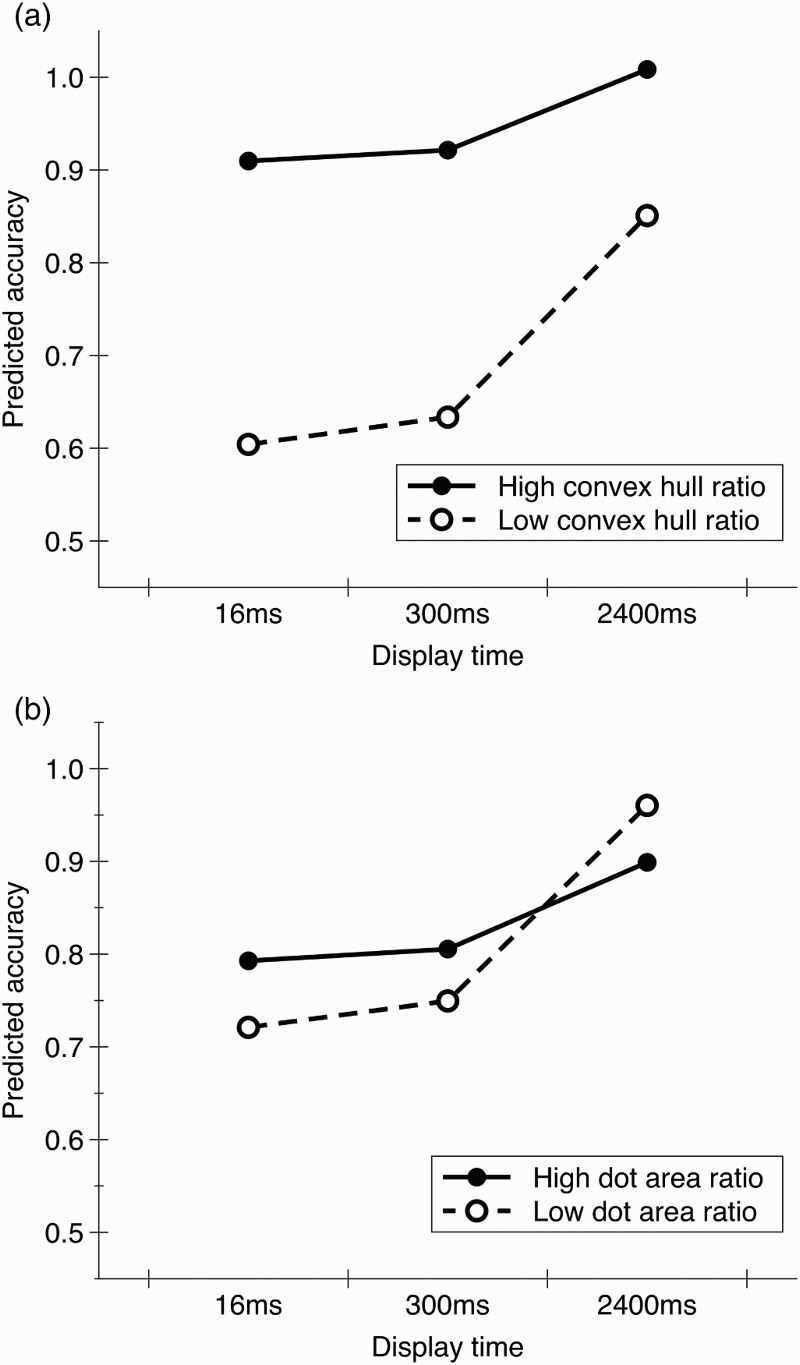
Predicted accuracy scores for each display time with (a) high (+1 *SD*) or low (−1 *SD*) values of convex hull ratio and mean values of dot area ratio and (b) high (+1 *SD*) or low (−1 *SD*) values of dot area ratio and mean values of convex hull ratio. In each case mean values of numerical ratio are used.

Overall the model was significant with *R* ^2^ = .72, *p* < .001. There were significant main effects of numerical ratio, *β* = .26, *t* = 7.29, *p* < .001, convex hull ratio, *β* = .58, *t* = 15.38, *p* < .001, and display time, *β* = .36, *t* = 9.75, *p* < .001. These were moderated by significant interactions: convex hull ratio × display time, *β* = −.15, *t* = −4.05, *p* < .001, dot area ratio × display time, *β* = −.14, *t* = −3.69, *p* < .001, convex hull ratio × dot area ratio, *β* = −.10, *t* = −2.75, *p* = .007, convex hull ratio × numerical ratio, *β* = −.23, *t* = −6.07, *p* < .001, a marginally significant interaction between convex hull ratio × numerical ratio × display time, *β* = −.08, *t* = −2.03, *p* = .043 and, as predicted, convex hull ratio × dot area ratio × display time, *β* = .11, *t* = 2.99, *p* = .003. Other effects were non-significant. Figure 2 displays predicted accuracy scores for high and low values of convex hull and dot area ratio for each display time.

To explore the three-way interaction between convex hull ratio, dot area ratio and display time, we investigated the relationship between accuracy and convex hull ratio and the relationship between accuracy and dot area ratio for each display time separately. There was a significant correlation between convex hull ratio and accuracy for each display time (16 ms: *r* = .75, *p* < .001, 300 ms: *r* = .76, *p* < .001, and 2400 ms: *r* = .51, *p* < .001). In contrast, the correlation between dot area ratio and accuracy was significant with a 16 ms display time, *r* = .51, *p* < .001, but there was no significant relationship for longer display times (300 ms: *r* = .15, *p* = .201, 2400 ms: *r* = −.04, *p* = .721).^3^ 3 This pattern of relationships remains the same if partial correlations are used. The correlation between convex hull ratio and accuracy controlling for dot area ratio is significant for each display time while the correlation between dot area ratio and accuracy controlling for convex hull ratio is significant only for the 16 ms display time but not for display times of 300 and 2400 ms. As in Experiment 1, the same pattern of effects is observed if performance is explored using simpler congruency effects. For this analysis all trials were categorized as congruent or incongruent for dot area and convex hull. An ANOVA with display time, dot area congruency and convex hull congruency as repeated-measures variables found a significant three-way interaction (*F*(2,38) = 5.2, *p* = .010). Dot area and convex hull congruency effects were calculated for each participant (congruent accuracy minus incongruent accuracy), and there was a significant negative correlation between dot area congruency effect and display time (*r* = −.34, *p* = .033), but no correlation between convex hull congruency effect and display time (*r* = .03, *p* = .84).


This result supports the findings of Experiment 1 by demonstrating that with longer display times, which reduce the difficulty of the task, adults were influenced by the convex hull ratio between the arrays but not the dot area ratio between the arrays. However, when task difficulty was increased by reducing the display time, dot area ratio also influenced adults’ performance. This provides further support to the proposal that convex hull information consistently affects participants’ judgements of numerosity, while dot area information only influences performance under certain conditions.

## General discussion

The dot comparison task is the predominant task used to assess the acuity of magnitude representations. Here, we show that the visual characteristics of dot arrays influence participants’ accuracy at making numerosity judgements, even when these visual cues are controlled. Specifically, there were significant effects of both convex hull ratio and dot area ratio on participants’ performance—indicating that their numerosity judgements were influenced by these visual cues. Importantly, however, the influence of these characteristics was dependent on the participants’ age and task design. With a moderate display time, dot area influenced the judgements of younger, but not older participants. When display time was reduced, even adult participants were susceptible to the influence of this visual cue. Convex hull information, on the other hand, appears to influence participants’ judgements more consistently—across age groups and display times. These findings have important methodological and theoretical implications, which are considered below.

These studies add further evidence that participants’ performance on dot comparison tasks is influenced by characteristics of the task (Clayton et al., 2015; Clayton & Gilmore, 2015; DeWind & Brannon, 2016; Gebuis & Reynvoet, 2012; Inglis & Gilmore, 2013, 2014; Price et al., 2012; Smets et al., 2015). In particular, we have shown that the visual characteristics of dot arrays affect the accuracy of numerosity judgements, and therefore performance on dot comparison tasks is not a pure measure of the acuity of ANS representations. Convex hull information, in particular, appears to consistently affect participants’ numerosity judgements. It is therefore particularly important to control for convex hull in the construction of dot arrays. However, commonly used methods for constructing dot arrays (e.g. Panamath, www.panamath.org), do not take account of convex hull and only control for dot size. Indeed, numerosity and convex hull are confounded in dot arrays produced by Panamath (Clayton et al., 2015; DeWind & Brannon, 2016). Given the evidence presented here, it is possible that performance on dot comparison tasks using these methods is based, in part, on judgements of convex hull and not solely numerosity. It is therefore not surprising that low correlations have been found between performance on dot comparison tasks using stimuli produced using these methods, compared to methods which also control for convex hull (Clayton et al., 2015; DeWind & Brannon, 2016; Smets et al., 2015).

Our findings also shed light on why some studies have failed to find congruency effects on participants’ dot comparison performance. Although the effect of convex hull ratio was consistent in all conditions of both Experiments 1 and 2, the effect of dot area ratio depended on both the age of the participants and the length of time that dot arrays were presented for. The use of different dot array construction methods, combined with differences in the task design and age of participants, may therefore account for the mixed evidence in the literature surrounding congruency effects. The lack of congruency effects in some studies does not, however, indicate that visual cues do not influence participants’ performance, rather it demonstrates that it is important to consider key visual cues such as convex hull.

Differences across studies in the influence of visual cues, combined with differences in participant and tasks characteristics (e.g. display time) may also shed light on why conflicting evidence exists regarding a correlation between dot comparison performance and mathematics achievement (De Smedt et al., 2013). Differences in the controls for visual characteristics of arrays raise a question about the appropriateness of combing across studies using different visual control protocols when trying to make sense of this conflicting evidence. Meta-analyses on this topic would therefore benefit from consideration of these features of studies.

Alongside these methodological implications, our findings also shed light on theoretical models of the processes involved in making numerosity comparisons. To date three alternative proposals have been put forward to account for an individual’s performance on dot comparison trials. According to the standard model (Barth et al., 2006; Halberda et al., 2008), performance on dot comparison trials is fully accounted for by differences in individuals’ underlying magnitude representations. Specifically, individual differences in accuracy on dot comparison trials arise because individuals differ in the precision of their magnitude representations—captured by differences in the Weber fraction (*w*). According to this account, visual characteristics of the arrays should not impact on performance and hence congruency effects should not be observed. Developmental differences in performance on dot comparison tasks are explained by changes in Weber fraction with age. This model cannot explain why we observed effects of visual cues, or why these effects change with age and differences in display time. Thus this model cannot account for our data.

Gebuis and Reynvoet (2012) set out an alternative account of numerosity comparison processes. They proposed that performance on dot comparison trials can be fully accounted for by considering the visual characteristics of arrays without recourse to abstract numerosity representations. Participants make judgements about numerosity on the basis of a combination of visual cues such as convex hull, dot size and density. This model is able to account for the presence of visual cue effects and predicts that the nature of visual cue effects will depend on the weighting given to different visual cues. This model can therefore account for our findings (Experiment 1) by assuming developmental changes in the salience of different visual cues, and therefore the weighting given to different visual cues in the estimates of numerosity. It would be assumed that dot area is a salient visual cue for younger participants but older participants give less weight to this feature when making magnitude judgements. Similarly, display time would be assumed to alter the salience of visual cues (Experiment 2), perhaps due to differences in the way these cues are perceived. Dot area would be assumed to be a more prominent cue with shorter exposure times but less weight is given to this cue with longer exposure times.

A final model of performance in magnitude comparison tasks takes account of both visual cues and the role of inhibition. The competing processes account (Gilmore et al., 2013) assumes that when participants are presented with dot comparison trials two competing processes influence the accuracy of their response. On the one hand, they can respond simply on the basis of the salience of different visual cues. However, if they successfully inhibit such a response then they respond on the basis of underlying numerosity representations. This model can account for the present findings by assuming that the success with which an individual can inhibit responses changes with development or exposure time. Dot area information is assumed to be easier to inhibit than convex hull information. Although older children and adults are able to inhibit this with sufficient exposure time, younger children, whose inhibition skills are known to be less well developed (Li, Hämmerer, Müller, Hommel, & Lindenberger, 2008), have difficulty inhibiting this cue. Similarly, when exposure time is short, even adults have difficulty inhibiting dot area information. The stability of convex hull congruency effects demonstrates that this cue is more difficult to inhibit regardless of age or display time, and thus participants are not always able to ignore this information and respond solely on the basis of numerosity representations.

Both the visual characteristics model (Gebuis & Reynvoet, 2012) and the competing processes model (Gilmore et al., 2013) are therefore able to account for the present findings. Further research is required in order to distinguish between these by exploring whether numerosity information has an independent influence on dot comparison performance over and above the influence of visual cues. Regardless, it is clear that participants’ performance on dot comparison tasks cannot be explained entirely by the role of the ANS. It is important, therefore, to better understand the features of tasks and individuals that influence numerosity judgements in order to discover the processes that underlie performance on these tasks. This, in turn, may reveal why there is conflicting evidence concerning a link between dot comparison performance and mathematics achievement, and whether evidence for a correlation represents a causal relationship.

## Disclosure statement

No potential conflict of interest was reported by the authors.
